# Prediction of risk and overall survival of pancreatic cancer from blood soluble immune checkpoint-related proteins

**DOI:** 10.3389/fimmu.2023.1189161

**Published:** 2023-05-15

**Authors:** Sai Pan, Wenting Zhao, Yizhan Li, Zhijun Ying, Yihong Luo, Qinchuan Wang, Xiawei Li, Wenjie Lu, Xin Dong, Yulian Wu, Xifeng Wu

**Affiliations:** ^1^ Center for Biostatistics, Bioinformatics and Big Data, The Second Affiliated Hospital and School of Public Health, Zhejiang University School of Medicine, Hangzhou, Zhejiang, China; ^2^ The Key Laboratory of Intelligent Preventive Medicine of Zhejiang Province, Hangzhou, Zhejiang, China; ^3^ Department of Surgical Oncology, The Affiliated Sir Run Run Shaw Hospital, Zhejiang University School of Medicine, Hangzhou, Zhejiang, China; ^4^ Department of Hepato-Pancreato-Biliary Surgery, The Second Affiliated Hospital, Zhejiang University School of Medicine, Hangzhou, Zhejiang, China

**Keywords:** soluble immune checkpoint-related protein, pancreatic cancer, immune subtype, survival, prediction model

## Abstract

**Background:**

Immune checkpoint inhibition holds promise as a novel treatment for pancreatic ductal adenocarcinoma (PDAC). The clinical significance of soluble immune checkpoint (ICK) related proteins have not yet fully explored in PDAC.

**Methods:**

We comprehensively profiled 14 soluble ICK-related proteins in plasma in 70 PDAC patients and 70 matched healthy controls. Epidemiological data of all subjects were obtained through structured interviews, and patients’ clinical data were retrieved from electronical health records. We evaluated the associations between the biomarkers with the risk of PDAC using unconditional multivariate logistic regression. Consensus clustering (k-means algorithm) with significant biomarkers was performed to identify immune subtypes in PDAC patients. Prediction models for overall survival (OS) in PDAC patients were developed using multivariate Cox proportional hazards regression. Harrell’s concordance index (C-index), time-dependent receiver operating characteristic (ROC) curve and calibration curve were utilized to evaluate performance of prediction models. Gene expressions of the identified ICK-related proteins in tumors from TCGA were analyzed to provide insight into underlying mechanisms.

**Results:**

Soluble BTLA, CD28, CD137, GITR and LAG-3 were significantly upregulated in PDAC patients (all *q* < 0.05), and elevation of each of them was correlated with PDAC increased risk (all *p* < 0.05). PDAC patients were classified into soluble immune-high and soluble immune-low subtypes, using these 5 biomarkers. Patients in soluble immune-high subtype had significantly poorer OS than those in soluble immune-low subtype (log-rank *p* = 9.7E-03). The model with clinical variables and soluble immune subtypes had excellent predictive power (C-index = 0.809) for the OS of PDAC patients. Furthermore, the immune subtypes identified with corresponding genes’ expression in PDAC tumor samples in TCGA showed an opposite correlation with OS to that of immune subtypes based on blood soluble ICK-related proteins (log-rank *p* =0.02). The immune-high subtype tumors displayed higher cytolytic activity (CYT) score than immune-low subtype tumors (*p* < 2E-16).

**Conclusion:**

Five soluble ICK-related proteins were identified to be significantly associated with the risk and prognosis of PDAC. Patients who were classified as soluble immune-low subtype based on these biomarkers had better overall survival than those of the soluble immune-high subtype.

## 1 Introduction

Pancreatic ductal adenocarcinoma (PDAC) remains one of the most lethal malignancies worldwide and ranks as the third leading cause of cancer death in the US (1, 2). The 5-year survival rate for PDAC remains low (less than 10%) over the past 4 decades, and it decreases to less than 1% in advanced patients (2, 3). Treatment for PDAC is currently limited to surgery and chemotherapy while efficacious strategies are desperately needed to improve the dismal prognosis of PDAC patients.

Molecular subtyping of PDAC is still in its infancy largely because there has been no clinically relevant molecular subtype that alternates treatment decision (4), unlike other solid tumors such as non-small cell lung cancer (5). *KRAS*, *TP53* and *CDKN2A* mutations, loss of *SMAD4* expression and *BRCA1/2* mutations were reported in PDAC patients (6). A transcriptomic subtyping was thus established by Bailey et al. (7), where PDAC patients were classified into four subtypes: squamous, pancreatic progenitor, immunogenic and ADEX (aberrantly differentiated endocrine exocrine). However, limited evidence of treatment responses based on these subtypes were reported in PDAC, especially for immunotherapy.

Immunotherapy in PDAC remains challenging due to multiple immune resistance mechanisms (8). Tumor-cell-intrinsic *KRAS* mutations could orchestrate a network of immune suppression in tumor microenvironment (TME) (9). Immune checkpoint blockade has been limited in the treatment of naïve PDAC patients due to factors such as the lack of activated T effector and TCR (T Cell Receptor) clonality (10), low MHC-I (Major Histocompatibility Complex-I) expression (11), low to moderate mutational burden limiting antigenic targets (12), and TME mediated suppression of T cell priming and function. Therefore, only small fraction (<1%) of PDAC patients responded to anti-PD1 for hypermutated MSI-H (Microsatellite Instability- High) tumors, let alone anti-CTLA4 or anti-PD-L1 therapies (13). Identifying the mechanisms of immune suppression and how to sensitize PDAC to immune checkpoint inhibitors are two major challenges of the immunotherapy in PDAC.

Soluble forms of membrane-bound receptors/ligands are generated by cleavage of membrane proteins or by alternative splicing from tumor cells/immune cells in TME or circulation (14). Soluble immune checkpoint proteins have been identified to be associated with advanced stage, survival and treatment response in multiple types of cancer (14). Soluble PD-1 and PD-L1 (sPD1 and sPD-L1) were identified as biomarkers of systemic inflammation in 41 advanced PDAC patients (15). Another study reported that soluble PD-1, PD-L1, BTLA, BTN3A1, and pan-BTN3As levels in plasma could predict survival in 59 PDAC patients, suggesting that these soluble immune checkpoint (ICK)-related proteins could be involved in anti-tumor immunity in PDAC (16). Studies also indicated that soluble ICK-related proteins might alter anti-tumor immunity by combining with corresponding immune checkpoint receptors/ligands, thereby influencing the outcomes of patients (14, 17). Nevertheless, the value of soluble ICK-related proteins in the diagnosis, prognosis and treatment of PDAC remains obscure. In present study, we implemented a two-stage study to systematically investigate the role of soluble ICK-related proteins in the prediction of cancer risk and overall survival in the patients of PDAC.

## 2 Materials and methods

### 2.1 Study design and participants

This study was approved by the Institutional Review Board of The Second Affiliated Hospital, Zhejiang University (SAHZU). At enrollment, a written informed consent was signed by all the participant and corresponding blood samples and clinical data were collected.

A schematic design of this study is illustrated in **Figure S1**. First, we systemically profiled the plasma levels of soluble ICK-related proteins in 70 PDAC patients and 70 matched healthy controls from SAHZU. The taxonomy of PDAC based on soluble ICK-related proteins was established. Second, we performed *in silico* functional validation of our taxonomy by exploring the transcriptomic levels of corresponding ICK genes in The Cancer Genome Atlas Pancreatic Adenocarcinoma (TCGA-PAAD) dataset.

PDAC patients were consecutively recruited from an ongoing pancreatic cancer cohort at SAHZU initiated in August, 2020. Patients who met the following criteria were included: 1. Pathologically (biopsy or fine-needle aspiration cytology) confirmed PDAC; 2. Informed consent or waiver of consent provided by each patient; 3. Follow-up information available. The exclusion criteria were as follows: 1. Patients had any prior treatment at the time of enrollment; 2. Non-PDAC or multiple cancer; 3. Failure to provide informed consent. Healthy controls without cancer diagnosis were recruited from an ongoing cohort on health individuals at SAHZU. To reduce the confounding effect, PDAC patients and healthy controls were matched with age (± 5 years) and sex using the propensity matching method (PSM) at the ratio of 1.

In addition, gene expression and phenotype data of PDAC tumor samples were retrieved from TCGA-PAAD cohort at GDC Data Portal (https://portal.gdc.cancer.gov/) (accessed July 6, 2022).

### 2.2 Baseline data collection

Epidemiological data were collected by SAHZU interviewers through face-to-face interview. Information on weight at 3 years before diagnosis (for patients) or enrollment (for controls), height, history of diabetes (yes/no), smoking status was recorded. Body Mass Index (BMI) was calculated by dividing weight by height squared (kg/m^2^), and it was categorized according to the WHO guideline: underweight and normal weight (< 25 kg/m^2^), overweight (≥ 25 kg/m^2^ but <30 kg/m^2^) or obese(≥30 kg/m^2^). A smoker was defined as an individual who had smoked at least 100 cigarettes in his or her lifetime; otherwise defined as a non-smoker. The clinical, pathological, and laboratory test data were retrieved from electronic medical records at SAHZU. Serum CA 19-9 levels at diagnosis (for patients) or at health checkups (for controls) were obtained. PDAC patients were staged by physicians in charge and pathologists according to the NCCN Guidelines for Pancreatic Adenocarcinoma (version 1.2022).

### 2.3 Sample collection and assessment of soluble ICK-related proteins

After the interview, 20 ml of venous blood from each participant was collected in EDTA (Ethylenediaminetetraacetic acid) tubes by phlebotomists and transported through cold chain to the laboratory in SAHZU. After centrifugation, plasma was aliquoted and stored under -80°C freezer until use. The plasma levels of 14 soluble ICK-related proteins (BTLA, LAG-3, GITR, IDO, PD-L2, PD-L1, PD-1, HVEM, TIM-3, CD28, CD27, CD80, CD137 and CTLA-4) were measured using multiplex assay kits (Thermo Fisher, USA) in a Luminex FLEXMAP 3D^®^ instrument (Luminex Corp, USA). Laboratory personnel were blinded to the case-control status of the samples. The manufacture’s protocol was followed for the assay procedure, which was described in our previous study (18). The lower limit of quantification (LLOQ) of each analyte was listed in **Table S1**.

### 2.4 Patient follow-up and outcomes

PDAC patients were regularly reviewed for vital status and disease progression every three months for the first two years, and twice a year thereafter. Death was confirmed on a death certificate from an attended hospital. Disease progression was measured by RECIST1.1 (Response Evaluation Criteria in Solid Tumours 1.1). Overall survival (OS) was defined as duration from the date of diagnosis to death of any cause or last follow-up. Progression-free survival (PFS) was defined as the duration from the date of diagnosis until disease progression or death, whichever occurred first. The loss to follow-up patients were censored. All patients were followed up for survival status until December, 2022.

### 2.5 Statistical analysis

Categorical variables were described as frequency and percentage [n (%)]. Continuous variables were described as mean (standard deviation, SD) or median [25th and 75th percentiles (Q1-Q3)]. Categorical variables were compared by Pearson’s χ^2^-test, and Wilcoxon rank-sum test or Student’s t-test was used to compare continuous variables. Unconditional logistic regression was performed to estimate the associations between each biomarker and PDAC risk with adjustment for age, sex, BMI, smoking status and history of diabetes. All biomarkers were considered as continuous variables and log-transformed to reduce skewness. False discovery rate (FDR) adjustment was applied to *p*-values (reported as *q*-values) to decrease the probability of Type I errors (19).

Unsupervised consensus clustering was employed on PDAC samples (both blood and tumor samples) using R package ConsensusClusterPlus (version 1.62.0) (20). K-means algorithm was used with 1,000 iterations to ensure the classification stability. The optimum number of clusters (k) was determined based on the proportion of ambiguously clustered pairs (PAC) and cumulative distribution function (CDF). Principal component analysis (PCA) was performed to show the distribution difference between the clusters. To reduce bias, for the soluble immune checkpoint-related proteins included in this study, we treated them as continuous variates in the consensus clustering analysis. For the covariates in the multivariate models, we used commonly accepted cut-off points.

Kaplan-Meier curve with log-rank test were used for comparing survival differences. Multivariate Cox proportional hazards regression models were developed by adding variables of interest sequentially to evaluate the effect on the outcomes of PDAC. Model 1 included clinical variables (sex, age, BMI, smoking status, diabetes and stage), followed by adding CA19-9 in model 2, or adding immune subtypes in model 3. The treatment (including surgery, chemotherapy, radiation, etc.) for PDAC subjects was not included in multivariate Cox model for its correlation with tumor stage. For instance, patients with resectable disease usually received surgery or surgery plus chemotherapy, whereas patients with locally advanced disease usually received palliative chemotherapy (21). Furthermore, as a sensitivity analysis, we included treatment information in the multivariate Cox model, and the results remain consistent. Time-dependent ROC (receiver operator characteristic) curve and Harrell’s concordance index (C-index) and were used to evaluate the discrimination of models. The calibration of each model was evaluated by calibration curve with 1,000 bootstrap resampling. The overall performance of the models was assessed by Brier score at a given time-point and integrated Brier score (IBS) at all available times (22). The gene expression data was downloaded from TCGA datasets. Cytolytic activity (CYT) was evaluated as the geometric mean of *GZMA* and *PRF1* expression according to a previous study (23).

We performed all statistical analysis in R (version 4.2.0). All statistical tests were two sided, and the significance level is 0.05.

## 3 Results

### 3.1 Participant characteristics

The baseline characteristics of all subjects, including 70 PDAC patients and 70 healthy controls was summarized in **Table 1**. All the participants of this study were Han Chinese. Over half of the participants were males. The mean ages of PDAC patients and healthy controls were 65.16 and 63.80 years, respectively. Over a third of participants were smokers with slightly more smokers in patients. Diabetes and serum CA19-9 were significantly different between PDAC patients and healthy controls (*p* < 0.001). Most patients were diagnosed at an advanced stage, in which more than half presented with metastatic disease. Serum CA19-9 levels were not elevated (below 37 U/mL) in 17 (24.29%) patients at diagnosis. Thirty-three (47.14%) patients died during the follow-up. The median follow-up time was 8.2 months (range: 0.3-28.1). Among all PDAC patients, 6 patients received radical surgery, 22 patients received radical surgery and chemotherapy, 28 patients received palliative chemotherapy, 9 patients received other treatment (palliative surgery, high-intensity focused ultrasound, ERCP (Endoscopic retrograde cholangiopancreatography) and PTCD (Percutaneous transhepatic bile duct drainage)), and 5 patients did not receive any treatment.

**Table 1:** Host characteristics of all participants.

Characteristics		Controls, n (%)	Cases, n (%)	*p*
n		70	70	
Age, mean (SD)		63.80 (9.62)	65.16 (10.13)	0.42
Sex				1.00
	Female	31 (44.29)	31 (44.29)	
	Male	39 (55.71)	39 (55.71)	
BMI, mean (SD)		24.25 (2.90)	23.55 (2.77)	0.15
Smoking				0.73
	No	42 (60.00)	39 (55.71)	
	Yes	28 (40.00)	31 (44.29)	
Diabetes				<0.001
	No	66 (94.29)	47 (67.14)	
	Yes	4 (5.71)	23 (32.86)	
Tumor location				–
	Head	–	39 (55.71)	
	Neck	–	5 (7.14)	
	Body	–	6 (8.57)	
	Tail	–	20 (28.57)	
T stage				–
	T1	–	2 (2.86)	
	T2	–	27 (38.57)	
	T3	–	23 (32.86)	
	T4	–	18 (25.71)	
N stage				–
	N0	–	39 (55.71)	
	N1	–	15 (21.43)	
	N2	–	16 (22.86)	
M stage				–
	M0	–	45 (64.29)	
	M1	–	25 (35.71)	
Stage				–
	Resectable	–	28 (40.00)	
	Locally advanced	–	17 (24.29)	
	Metastatic	–	25 (35.71)	
CA19-9 (U/ml)				<0.001
	Normal (<37 U/ml)	70 (100.0)	17 (24.29)	
	Elevated (≥37 U/ml)	0 (0.0)	53 (75.71)	
Treatment				NA
	Radical surgery only	–	6 (8.60)	
	Radical surgery plus chemotherapy	–	22 (31.40)	
	Palliative chemotherapy	–	28 (40.00)	
	*Others	–	9 (12.90)	
	None	–	5 (7.10)	

*Others indicate palliative surgery, high-intensity focused ultrasound, ERCP and PTCD.

SD, standard deviation.

### 3.2 Associations between soluble ICK-related proteins and PDAC risk

The distributions of 14 soluble immune checkpoint-related proteins (median and Q1-Q3) were listed in **Table 2**. Soluble HVEM and PD-L1 were excluded from the subsequent analysis because most of the measurements were below the LLOQ.

**Table 2:** Plasma levels of soluble immune checkpoint-related proteins in cases and controls.

Markers	Controls (n=70)	Cases (n=70)	*p*	*q^b^*
	Median (IQR) pg/ml	Median (IQR) pg/ml		
BTLA	508.53 (306.86-748.43)	642.74 (409.05-1009.30)	**0.02**	**0.04**
CD27	103.02 (60.39-177.17)	127.48 (49.39-358.83)	0.13	0.19
CD28	32.47 (29.67-52.20)	54.84 (32.47-125.98)	**4.20E-04**	**5.04E-03**
CD80	129.39 (78.23-239.56)	131.73 (53.42-356.89)	0.54	0.59
CD137	40.82 (28.77-63.13)	68.61 (36.58-124.93)	**4.72E-03**	**1.42E-02**
CTLA-4	20.00 (14.83-25.30)	27.23 (13.09-47.52)	0.09	0.15
HVEM^a^	15.01 (15.01-15.01)	15.01 (15.01-15.01)	–	–
GITR	21.48 (13.01-34.03)	33.69 (21.21-51.01)	**2.90E-03**	**1.16E-02**
IDO	23.73 (14.43-31.44)	24.28 (16.08-47.82)	0.19	0.23
LAG-3	15.11 (11.02-22.24)	22.33 (14.68-37.26)	**1.61E-03**	**9.66E-03**
PD-1	51.49 (31.25-80.63)	55.86 (33.60-110.77)	0.18	0.23
PD-L1^a^	3.49 (3.49-3.49)	3.49 (3.49-3.49)	–	–
PD-L2	2990.45 (2044.44-3643.34)	2849.28 (2051.36-5299.90)	0.61	0.61
TIM-3	622.85 (460.61-923.06)	807.28 (435.30-1520.76)	0.06	0.12

a Soluble HVEM and PD-L1 were not included in the subsequent analysis for most measurements below the LLOQ.

b FDR-correction was applied.

The bold values means significant p value.

**Figure 1:**
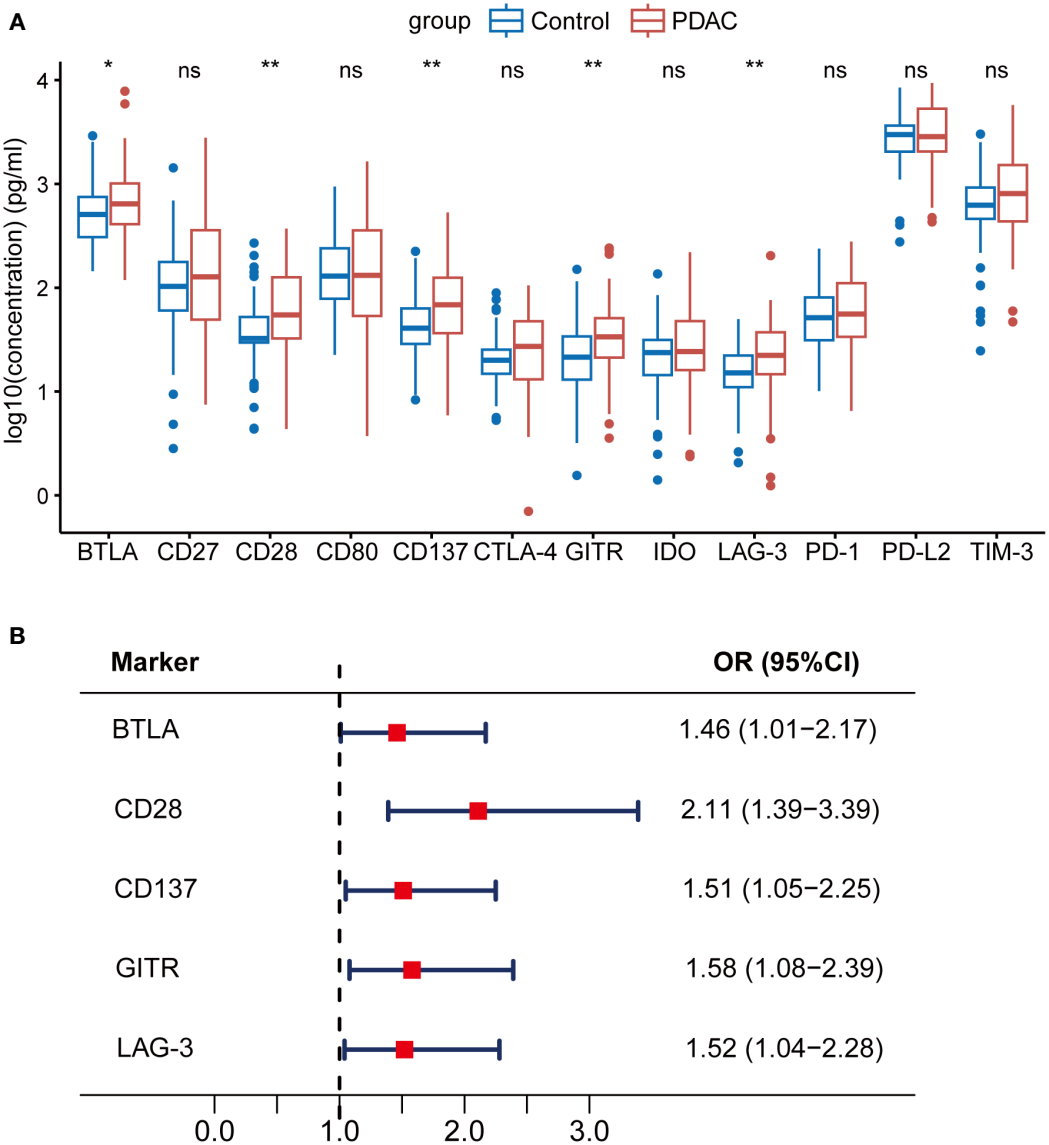
Associations between soluble immune checkpoint-related proteins and PDAC risk. sBTLA, sCD28, sCD137, sGITR, and sLAG-3 were significantly increased in PDAC patients compared to healthy controls. **(A)** Comparison of 12 soluble immune checkpoint-related proteins between PDAC patients (n=70) and healthy controls (n=70). **(B)** Forest plot of multivariable-adjusted ORs of the 5 biomarkers in unconditional logistic regression analysis. The vertical dash line indicates an OR of 1.0. The solid square with horizontal lines corresponding to the ORs and 95% CIs. PDAC, pancreatic ductal adenocarcinoma; OR, odds ratio; CI, confidence interval. ∗*q <*0.05; ∗∗*q <*0.01; ‘ns’ indicated not significant.

We identified that sBTLA, sCD28, sCD137, sGITR, and sLAG-3 were significantly increased in PDAC patients as compared to those in healthy controls (**Figure 1A**, **Table 2**, all *q* < 0.05). Logistic regression adjusted for age, sex, BMI, smoking and diabetes indicated that these 5 biomarkers were significantly associated with PDAC risk (**Figure 1B**, **Table S2**, all *q* < 0.05). Among them, sCD28 was the most significantly associated biomarker with PDAC risk (Odds ratio (OR) = 2.11, 95% CI: 1.39-3.39).

### 3.3 Association of soluble ICK-related proteins taxonomy and prognosis of PDAC

**Figure 2:**
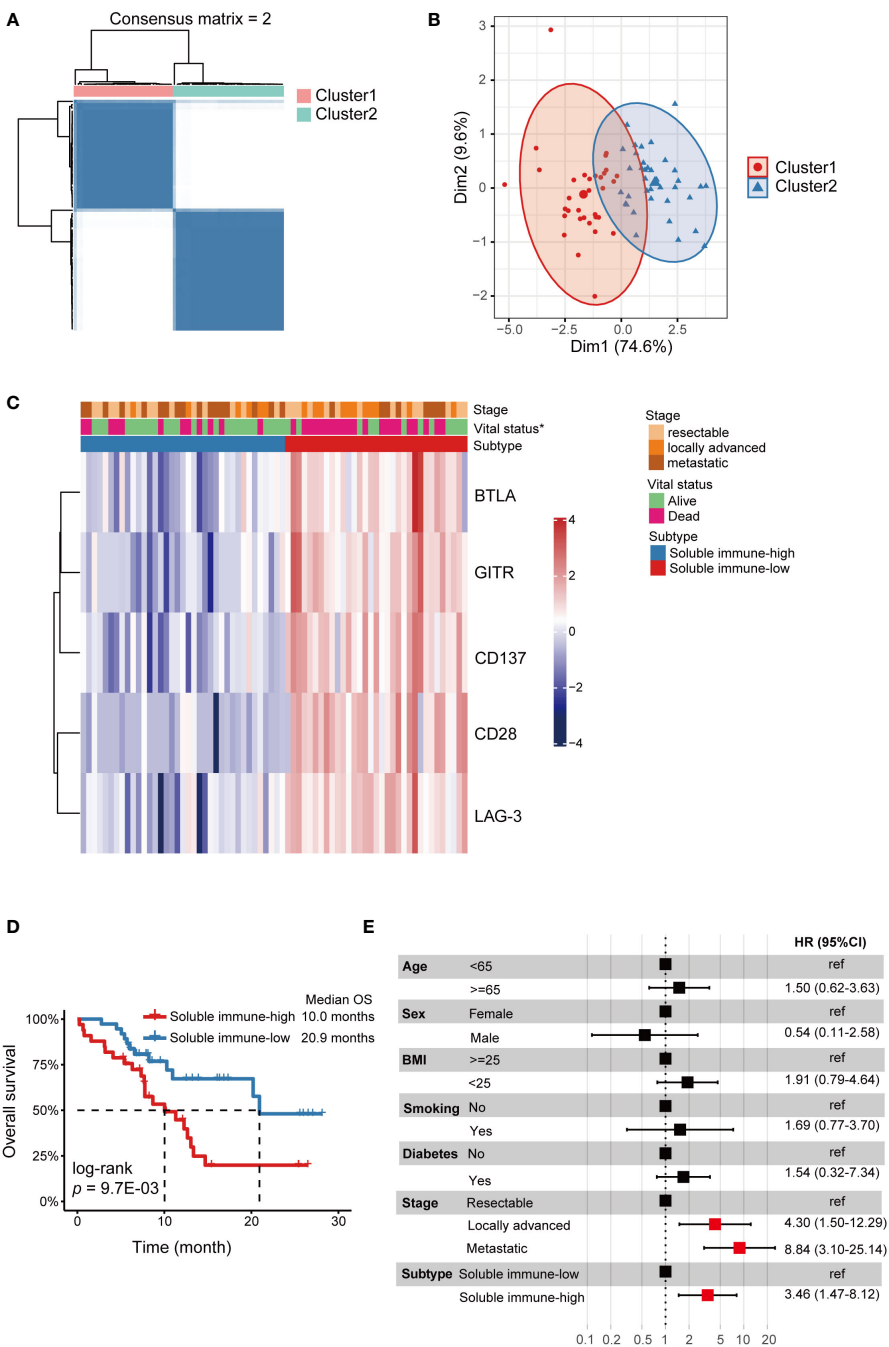
Identification of soluble immune checkpoint-related proteins-based subtypes in PDAC. Soluble immune subtypes based on soluble immune checkpoint-related proteins were associated with outcomes of PDAC patients. **(A)** Consensus clustering matrix for *k*=2 based on the plasma levels of soluble immune checkpoint-related proteins by unsupervised consensus clustering method (k-means). **(B)** PCA showed obvious segregation between the two clusters. **(C)** Heatmap for the associations between the clusters and clinicopathological characteristics. Subtype, age, BMI, smoke, tumor location, N stage, M stage, stage and vital status were used as annotations. ∗*p <*0.05. **(D)** Kaplan-Meier curve showed that OS of the soluble immune-high subtype patients was significantly poorer than those in the soluble immune-low subtype. **(E)** Forest plot of multivariate Cox regression analysis. Covariates were age, sex, BMI, smoking, diabetes, stage and subtype. The vertical dash line indicates an HR of 1.0. The solid square with horizontal lines corresponding to the HRs and 95% CIs. PDAC, pancreatic ductal adenocarcinoma; PCA, principal component analysis; OS, overall survival; HR, hazards ratio; CI, confidence interval.

Unsupervised consensus clustering was performed in PDAC patients based on the levels of the 5 identified biomarkers (sBTLA, sCD28, sCD137, sGITR and sLAG-3). We found that when k = 2, the slope of the CDF curve was flat and relative area change was large (**Figure 2A**, **Figure S2**); PCA outputted k=2. Thus, we classify the PDAC patients into 2 clusters. There were 33 and 37 cases in cluster 1 and 2, respectively. PCA analysis showed obvious segregation between the two clusters (**Figure 2B**). The two clusters also had striking different profiles of the 5 biomarkers. Cluster 1 displayed a higher relative abundance of the biomarkers compared to cluster 2 (**Figure 2C**). Thus, we designated these two clusters as soluble immune-high (cluster 1) and soluble immune-low (cluster 2) subtype. The relationships between the immune subtype and clinicopathologic characteristics were investigated, but there were no significant differences other than vital status (**Figure 2C**, **Table S3**). We also compared the OS between the two soluble immune subtypes, and showed that the OS was remarkably shorter in soluble immune-high subtype than that of soluble immune-low subtype (log-rank *p* = 9.7E-03) (**Figure 2D**). Furthermore, the multivariate Cox regression model indicated that soluble immune subtype was an independent predictor of OS (Hazard ratio (HR) = 3.46, 95% CI: 1.47-8.12) (**Figure 2E**).

### 3.4 Predictive models for OS in PDAC

**Figure 3:**
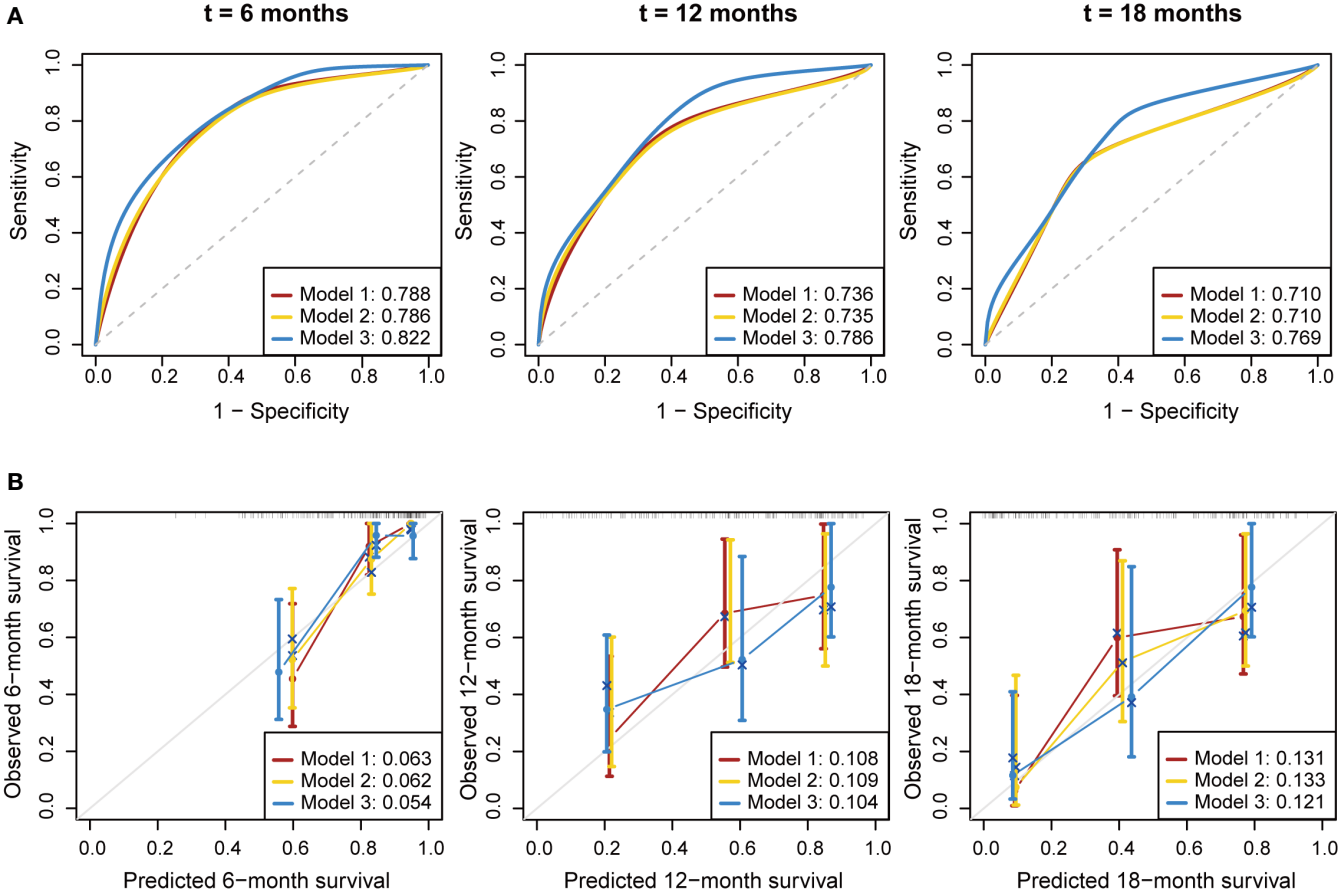
Performance evaluation for the predictive models. Multivariate Cox proportional hazards models were established to predict the overall survival of PDAC patients. **(A)** Time-dependent ROC curve of the three prognostic models at 6, 12 and 18 months. **(B)** Calibration curve of the three prognostic models at 6, 12 and 18 months. Bier score was used to quantify the prediction error. The gray line indicated the ideal reference line. ROC, receiver operating characteristic; AUC, area under curve.

We developed three multivariate Cox proportional hazards regression models to predict the OS of PDAC patients (**Table 3**). In model 1 (clinical variables only), locally advanced and metastatic patients showed significantly poorer OS compared to resectable patients (HR = 5.12, 95% CI: 1.87-13.99; HR = 6.24, 95% CI: 2.37-16.42, respectively). In model 2 (clinical variables + CA19-9), CA19-9 elevation was not statistically associated with OS. In model 3 (model 1 + soluble immune subtypes), patients of soluble immune-low subtype showed significantly better OS compared to patients of soluble immune-high subtype (HR = 3.46, 95% CI: 1.47-8.12). As shown in **Figure 3A** and **Table 4**, model 3 demonstrated better discrimination than the other two models. The C-index of model 3 was the highest (0.809, 95% CI: 0.733-0.885), followed by model 2 (0.764, 95% CI: 0.686-0.842), and model 1 (0.763, 95% CI: 0.690-0.836). The area under the curve (AUC) of model 3 was also the highest among the three models, which was 0.822, 0.786 and 0.769 at 6, 12 and 18 months, respectively. The calibration curve of model 3 for the prediction of 6-, 12- and 18-month OS showed promising agreement between the predicted and actual results (**Figure 3B**). Brier score of model 3 was the lowest among the three models at 6, 12 and 18 months, and IBS was the same (**Table 4**). Collectively, these results indicated that model 3 outperformed the models without soluble immune subtype as covariate and thus could be a useful predictive model for OS in PDAC patients.

**Table 3:** Multivariate Cox regression models in prediction of overall survival in PDAC patients.

Variables	Model 1	Model 2	Model 3
	HR (95% CI)	HR (95% CI)	HR (95% CI)
Age			
<65	1 (reference)	1 (reference)	1 (reference)
>=65	2.00 (0.85-4.71)	2.20 (0.91-5.36)	1.50 (0.62-3.63)
Sex			
Female	1 (reference)	1 (reference)	1 (reference)
Male	0.47 (0.10-2.14)	0.44 (0.09-2.04)	0.54 (0.11-2.58)
BMI			
<25	1 (reference)	1 (reference)	1 (reference)
>=25	1.40 (0.62-3.17)	1.54 (0.66-3.59)	1.91 (0.79-4.64)
Diabetes			
No	1 (reference)	1 (reference)	1 (reference)
Yes	1.62 (0.72-3.62)	1.60 (0.72-3.56)	1.69 (0.77-3.70)
Smoking			
No	1 (reference)	1 (reference)	1 (reference)
Yes	1.67 (0.36-7.76)	1.84 (0.39-8.69)	1.54 (0.32-7.34)
Stage			
Resectable	1 (reference)	1 (reference)	1 (reference)
Locally advanced	5.12 (1.87-13.99)	5.10 (1.86-14.00)	4.30 (1.50-12.29)
Metastatic	6.24 (2.37-16.42)	6.87 (2.52-18.70)	8.84 (3.10-25.14)
CA19-9			
Normal	–	1 (reference)	–
Elevated	–	1.47 (0.55-3.94)	–
Subtype			
Soluble immune-low	–	–	1 (reference)
Soluble immune-high	–	–	3.46 (1.47-8.12)

Model1: epidemiology variables, Model 2: epidemiology variables + CA19-9, Model 3: epidemiology variables + immune subtype.

**Table 4:** Predictive evaluation metrics of different models.

Metrics	Model 1	Model 2	Model 3
C-index (95%CI)	0.763 (0.690-0.836)	0.764 (0.686-0.842)	0.809 (0.733-0.885)
AUC			
6 months	0.788	0.786	0.822
12 months	0.736	0.735	0.786
18 months	0.710	0.710	0.769
IBS	0.156	0.157	0.145
Brier score			
6 months	0.063	0.062	0.054
12 months	0.108	0.109	0.104
18 months	0.131	0.133	0.121

*model 1 (clinical variables only, including sex, age, BMI, smoking status, diabetes and stage), model 2 (clinical variables + CA19-9), model 3 (model 1 + soluble immune subtypes).

### 3.5 Identification of distinct immune checkpoints subtypes in TCGA-PAAD cohort

**Figure 4:**
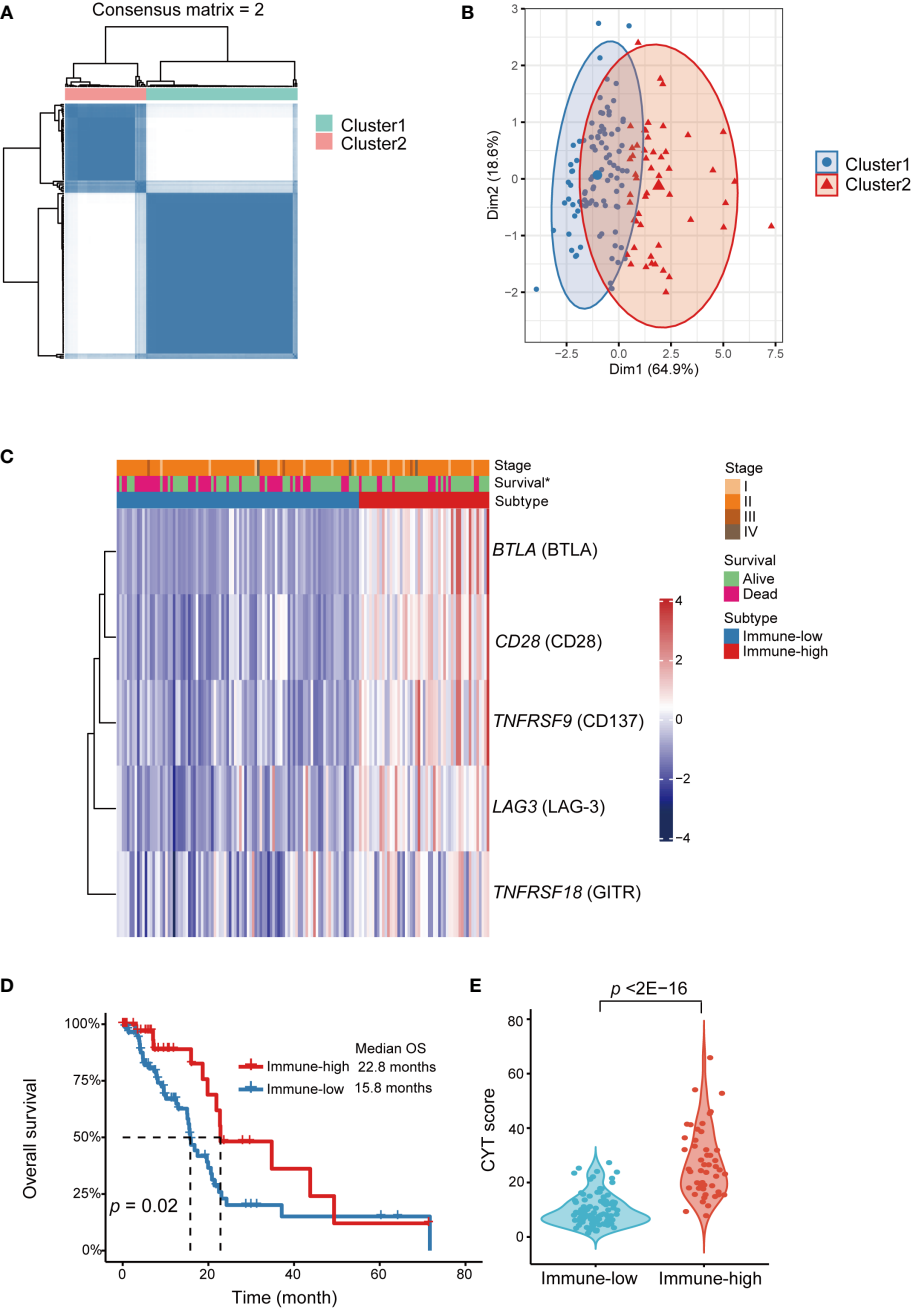
Identification of immune checkpoint genes expression subtypes in TCGA-PAAD cohort. Functional explorations of our immune subtypes applying transcriptomic data from TCGA PAAD dataset. **(A)** Consensus clustering matrix for *k*=2 based on the expression levels of immune checkpoint by unsupervised consensus clustering method (k-means). **(B)** PCA showed obvious segregation between the two clusters. **(C)** Heatmap for the associations between the clusters and clinicopathological characteristics. Subtype, age, sex, smoking, diabetes, stage, grade and vital status were used as annotations. ∗*p <*0.05. **(D)** Kaplan-Meier curve showed that OS of the immune-low subtype patients was significantly poorer than those in the immune-high subtype. **(E)** Comparison of CYT score between two subtypes. PDAC, pancreatic ductal adenocarcinoma; PCA, principal component analysis; OS, overall survival; CYT, cytolytic activity.

Immune checkpoint genes’ expression in TCGA-PAAD cohort was employed to further validate and explore the immune subtypes in PDAC. The demographic and clinical information of PDAC samples were listed in **Table S4**. Based on the expression levels of *BTLA*, *CD28*, *TNFRSF9* (gene coding for CD137)*, TNFRSF18* (gene coding for GITR) and *LAG3*, two different patterns were determined by unsupervised consensus clustering (**Figure S3**), including immune-low (cluster 1, n = 95) and immune-high (cluster 2, n = 51) subtypes (**Figures 4A–C**, **Table S5**). Interestingly, patients in the immune-low subtype demonstrated significantly poorer OS compared to the patients in the immune-high subtype (log-rank *p* = 0.02) (**Figure 4D**). Besides, we found that the CYT score was significantly higher in immune-high subtype than that in immune-low subtype (*p* < 2E-16) (**Figure 4E**).

## 4 Discussion

We identified immune subtypes of PDAC based on soluble ICK-related proteins, established survival predictive models combining the biomarkers and clinical variables, and successfully applied in the PDAC patients. We found that soluble BTLA, CD28, CD137, GITR, and LAG-3 were significantly associated with PDAC risk, and these biomarkers could further classify PDAC patients into soluble immune-high and soluble immune-low subtypes. Our multivariate model including the soluble immune subtypes performed better than a similar model using CA19-9 in predicting overall survival of the PDAC patients. Functional analysis employing transcriptomic data from TCGA validated that the expression of the five ICK genes could also stratify the PDAC tumor samples into two clusters. The patients with high soluble immune checkpoint-related proteins levels (soluble immune-high) had significantly poorer OS than the patients with low levels (soluble immune-low). Interestingly, the association with OS in tumoral transcriptomic levels of corresponding genes was opposite from what was observed in the blood soluble protein levels, indicating the distinct roles of soluble ICK-related proteins versus tumoral ICK related proteins in the development of PDAC. Our findings illustrated the values of soluble ICK-related proteins in the risk and prognosis prediction of PDAC.

To our knowledge, soluble ICK-related proteins have not been jointly assessed for their combined contribution to PDAC patients’ survival. Our results defined two robust soluble immune subtypes based on 5 soluble ICK-related proteins, which could successfully predict survival of PDAC patients in the multivariate Cox regression model. The 5 biomarkers were all high in soluble immune-high subtype and were all low in soluble immune-low subtype, which suggested a common function in interfering anti-tumor immunity and regulation of these immune proteins in PDAC. In transcriptomic subtyping, the CYT score in the immune-low subtype was significantly lower compared with that in the immune-high subtype, indicating that tumors in the immune-low subtype lack CD8^+^ T cells or have CD8^+^ T cell anergy (23). Thus, the prognosis of the patient with immune-low subtype was poor. Interestingly, our cohort showed an opposite result where soluble immune-high subtype had poorer OS compared to soluble immune-low subtype. This discrepancy was also observed in solid tumor patients treated with anti-PD-1/PD-L1, where plasma sBTLA, sCD28, sGITR and sLAG-3 levels were significantly lower in the durable benefit group, but the corresponding genes expression levels were higher than the non-durable clinical benefit group (24). Furthermore, the upregulated genes expression group showed significantly increased fractions of CD8^+^ T cells (24). Soluble ICK-proteins could competitively interfere with the interactions of their corresponding receptors/ligands in the membrane of immune/tumor cells, which could subsequently alter the anti-tumor immunity and cancer outcomes (12, 15). Further studies focus on the blood, tumoral and immune cellular levels of immune checkpoint-related proteins in a same cohort is warranted to validate our findings and also to explore the potential mechanisms underlying the discrepancy.

CD28 is a major co-stimulatory receptor constitutively expressed on naïve T cells that induces T cell differentiation and proliferation upon ligation by CD80 or CD86 on antigen-presenting cells (25). sCD28 could be derived from alternatively spliced transcripts in resting T cells. One of the sCD28 variants has intensities similar to membrane CD28, suggesting it may participates in immune regulation (26). We found that sCD28 was most strongly associated with PDAC risk, and high plasma sCD28 concentrations associated with poor OS in PDAC patients. This was consistent with findings in hepatocellular carcinoma (HCC), in which high baseline levels of sCD28 predicted a significantly greater HCC cumulative rate (27). Whereas treatment-induced survival benefit was strongly correlated with decreased sCD28 levels (28). Also, sCD28 levels were higher in breast cancer patients than in healthy controls (29). sCD28 was found to inhibit CD28 signaling by competing for B7 ligands, thereby impairing T-cell activation (30, 31). Besides, sCD28 could lead to dendritic cells-induced IL-6 production in *in vitro* cultures (32). IL-6 was a driver of tumorigenesis and metastasis in PDAC (33). Overall, sCD28 is a useful biomarker for clinical outcome in PDAC patients, though more validation is required.

CD137 is an important costimulatory molecule expressed on the surface of activated CD8^+^ T cells (34). The interaction of CD137/CD137L led to T cell activation and survival, thereby enhancing antitumor immunity. CD137 agonist antibodies had shown therapeutic effects in pancreatic cancer alone or in combination with other agents including PD-1 checkpoint inhibitors (35–37). In our study, high levels of sCD137 were associated with increased PDAC risk and shorter OS in PDAC patients. Previous studies had reported that sCD137 was overexpressed in NSCLC patients and CLL patients compared with the control group (38, 39). Moreover, uveal melanoma patients with fast-progressive disease had higher levels of sCD137 as compared to slow progressors and long survivors (40). Soluble CD137 was generated by alternately splicing of *TNFRSF9* (Tumor Necrosis Factor Receptor Superfamily Member 9) in activated T cells (41), which could not only block CD137-CD137L interactions but directly suppress effector T cells via CD137L, thereby preventing co-stimulation of T lymphocytes (42). In addition, Labiano et al. (43) found that cell lines from hepatocellular tumors, lung, renal, and melanoma selectively expressed sCD137 but not membrane-bound CD137 (mCD137) under hypoxia condition, suggesting sCD137 was beneficial for tumor survival. Taken together, the results indicated that sCD137 could be an inhibitor of effector T cells, which could help immune evasion and survival of tumor.

LAG-3, is one of suppressive immune checkpoints expressed on the surface of T cells, which has been reported to be associated with reduced survival in pancreatic cancer (44). LAG-3 antagonist in combination with other immunologic agents increased antitumor immunity and achieved durable remission in PDAC (37). After T cell activation, LAG-3 cleavage was increased, resulting in the release of sLAG-3 (45). Our results indicated that high levels of sLAG-3 were associated with increased risk and reduced OS of PDAC patients. Similar findings on the associations of sLAG-3 with poor survival in CLL patients had been reported (46). Also, high sLAG-3 levels were also found to be predictive of poor prognosis in several solid tumors, including melanoma (47), NSCLC (48), and HNSCC (49). However, in gastric cancer, sLAG-3 could positively regulate and enhance the anti-tumor immunity of CD8^+^T cells and secretion of IL-12 and IFN-γ, thereby improve the survival of patients (50). The discrepancy may stem from the distinct immune contextures and mechanisms among different cancer types (51).

GITR is another novel stimulatory immune checkpoint in NK cells and T cells, whose agonism could promote anti-tumor immunity by reducing the regulatory T cells and stimulating the proliferation and activation of effector T cells (52). sGITR elevation was associated with increased risk and reduced OS of PDAC in our study. The result was partially supported by a study indicating high GITR expression in tumor was associated with poor relapse-free survival in a cohort of breast cancer patients (53). Our previous study also indicated that sGITR was associated with increased risk of biochemical recurrence and progression of prostate cancer (54). Therefore, GITR has gain increasing interest of research as a promising target of immunotherapy (55). Also, BTLA is an important co-inhibitory molecule on the surface of antigen presenting cells, which could interact with its ligand HVEM to suppress the T cell activity (56). sBTLA is identified correlating with increased cancer risk and reduced OS of PDAC in our study. High level of sBTLA was found to be associated with reduced survival in ccRCC and pancreatic cancer (16, 18), which was consistent with our findings. sBTLA could interact with membrane HVEM on tumor cells, thereby blocking BTLA/HVEM axis and promoting tumor growth via the ERK1/2 pathway (57). This study partially explained the mechanism of sBTLA associated poor survival in PDAC patients.

Our study has several strengths like multiplex soluble ICK-related proteins profiling, establishing the immune subtypes and prediction models of PDAC based on soluble ICK-related proteins, and a novel approach that significantly increased predictivity of survival in PDAC patients. However, the study has several limitations. First, our 70 cases of PDAC sample size were relatively small although the study was well designed and all the participants were strictly matched to avoid potential confounding factors. Second, we did not evaluate corresponding genes’ expression in tumors and peripheral blood mononuclear cells. Instead, we applied the immune checkpoint and cytolytic activity genes expression in TCGA database to decipher potential mechanisms. Third, the study was a retrospective study in a single institution, more independent validations were required.

## 5 Conclusion

In this study, we established soluble immune subtypes of PDAC based on soluble ICK-related proteins for the first time. We further established predictive model using the soluble immune subtypes and clinical variables. PDAC patients who were classified as soluble immune-low subtype had better OS than those of the soluble immune-high subtype. Our findings indicated that immune-high PDAC patients may be more suitable for immune checkpoint blockade therapy. Future studies may apply the immune subtypes in prospective settings to examine the accuracy of survival predictions and treatment outcomes of immunotherapy of PDAC patients.

## Data availability statement

The original contributions presented in the study are included in the article/**Supplementary Material**. Further inquiries can be directed to the corresponding authors.

## Ethics statement

The studies involving human participants were reviewed and approved by Institutional Review Board of The Second Affiliated Hospital, Zhejiang University. The patients/participants provided their written informed consent to participate in this study.

## Author contributions

XW involved in conception and design; SP, QW and WZ involved in development of methodology; XL, WL, XD, YW and SP participated in acquisition of data (acquired and managed patients, provided facilities, etc.); SP, YzL, ZY, YhL and YW involved in analysis and interpretation of data (e.g., statistical analysis, biostatistics, computational analysis); QW, SP, WZ and XW drafted the manuscript and all the authors participant in the revision of the manuscript; XW, YW involved in study supervision.
