# Supplementary material for: Prediction of risk and overall survival of pancreatic cancer from blood soluble immune checkpoint-related proteins
Source: Front Immunol. 2023 May 15;14:1189161. doi: 10.3389/fimmu.2023.1189161 (PMC10225568; doi:10.3389/fimmu.2023.1189161)
Supplement: Supplementary file 1 [file DataSheet_1.docx]

Supplementary Material

Prediction of risk and overall survival of pancreatic cancer from blood soluble immune checkpoint-related proteins

Sai Pan^1,4†^, Wenting Zhao^1,4†^, Yizhan Li^1,4^, Zhijun Ying^1,4^, Yihong Luo^1,4^, Qinchuan Wang^1,2,4^, Xiawei Li^3^, Wenjie Lu^3^, Xin Dong^3^, **Yulian Wu**^3*^, **Xifeng Wu^1,4*^**

*Co-correspondence:
Xifeng Wu, MD, PhD, Center for Biostatistics, Bioinformatics and Big Data, The Second Affiliated Hospital and School of Public Health, Zhejiang University School of Medicine, 866 Yuhangtang Rd, Hangzhou, 310058, PR China, Tel: +17132480685, email at [xifengw@zju.edu.cn](mailto:xifengw@zju.edu.cn)

Yulian Wu, MD, Department of Hepato-Pancreato-Biliary Surgery, The Second Affiliated Hospital, Zhejiang University School of Medicine, 866 Yuhangtang Rd, Hangzhou, 310058, PR China, Tel: + 86-0571-87784604, email at [yulianwu@zju.edu.cn](mailto:yulianwu@zju.edu.cn)

^†^These authors have contributed equally to this work

# Supplementary Figures and Tables

## Supplementary Figures


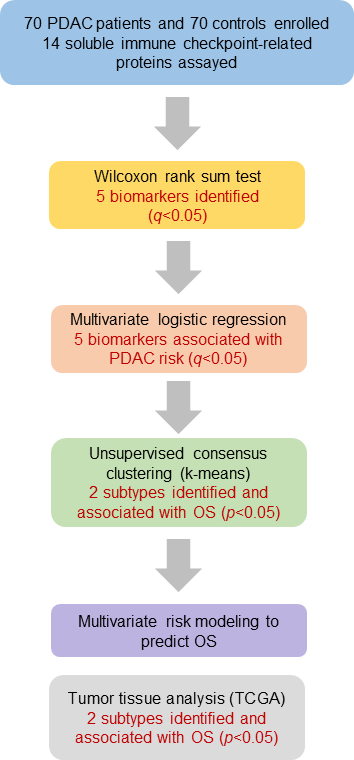


**Supplementary Figure S1.** A schematic design of the study.


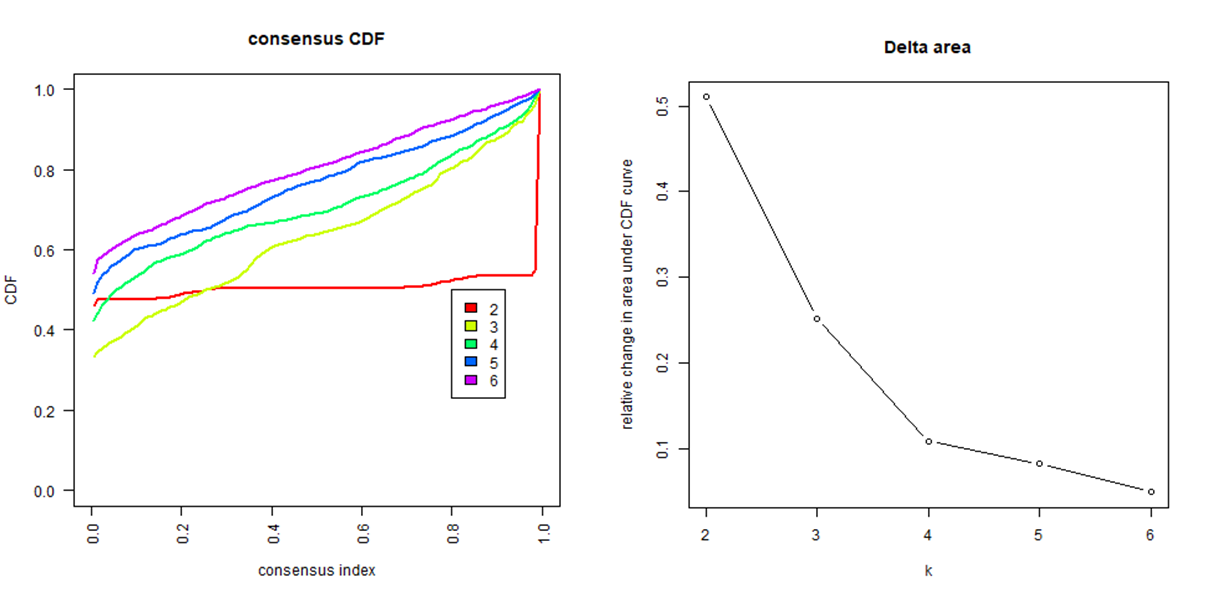


**Supplementary Figure S2.** Consensus cumulative distribution function (CDF) and delta area plot indicated the optimal number of clusters was two in PDAC patients.


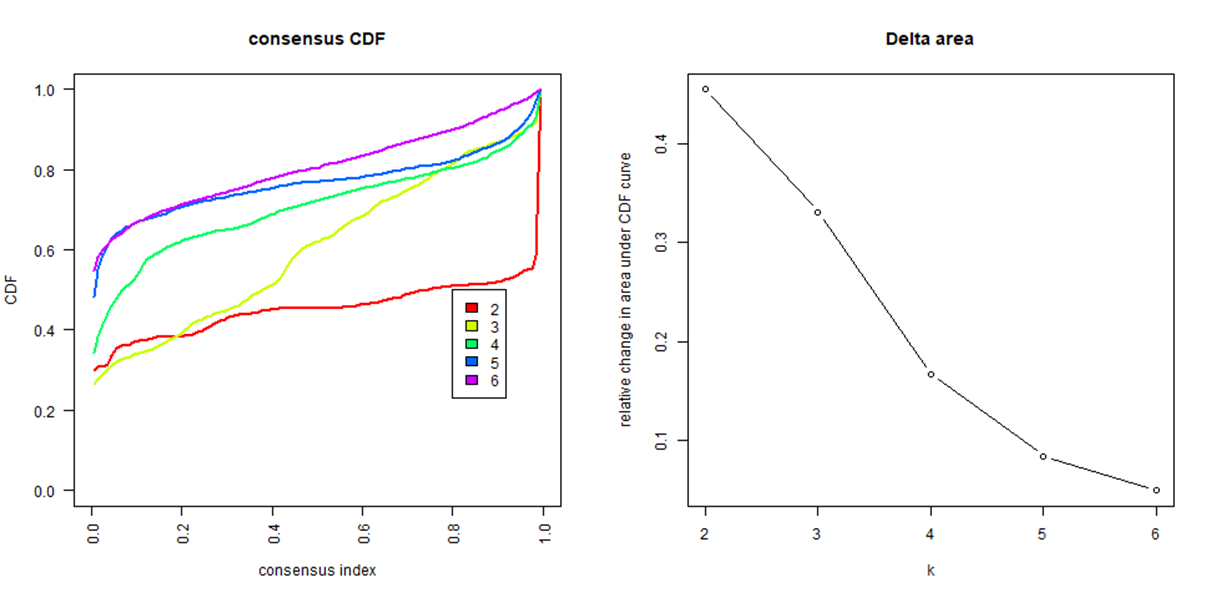


**Supplementary Figure S3**. Consensus cumulative distribution function (CDF) and delta area plot indicated the optimal number of clusters was two in PDAC samples of TCGA-PAAD cohort.

## Supplementary Tables

**Supplementary Table S1.** Lower limits of quantification (LLOQ) of each analyte of the panel (data from ThermoFisher†).

| **Analyte** | **LLOQ (pg/ml)** |
| --- | --- |
| BTLA | 126.00 |
| CD27 | 4.83 |
| CD28 | 32.47 |
| CD80 | 42.00 |
| CD137 (4-1BB) | 15.14 |
| CD152 (CTLA-4) | 8.57 |
| GITR | 21.22 |
| HVEM | 15.01 |
| IDO | 3.66 |
| LAG-3 | 12.00 |
| PD-1 | 6.98 |
| PD-L1 | 3.49 |
| PD-L2 | 45.00 |
| TIM-3 | 59.59 |

† Immuno-Oncology Checkpoint 14-Plex Human ProcartaPlex™ Panel 1, https://www.thermofisher.com/order/catalog/product/EPX14A-15803-901

**Supplementary Table S2.** Unconditional logistic regression of soluble immune checkpoint-related proteins in PDAC patients and healthy controls.

| **Markers** |  | **Univariate^a^** | | |  | **Multivariate^a^** | | |
| --- | --- | --- | --- | --- | --- | --- | --- | --- |
|  |  | **OR (95%CI)^a^** | ***p*** | ***q^b^*** |  | **OR (95%CI)^a^** | ***p*** | ***q^b^*** |
| BTLA |  | 1.48 (1.05-2.15) | **0.03** | **0.03** |  | 1.46 (1.01-2.17) | **0.05** | **0.05** |
| CD28 |  | 2.16 (1.46-3.34) | **2.32E-04** | **1.16E-03** |  | 2.11 (1.39-3.39) | **9.35E-04** | **4.68E-03** |
| CD137 |  | 1.64 (1.16-2.39) | **6.90E-03** | **0.01** |  | 1.51 (1.04-2.25) | **0.03** | **0.05** |
| GITR |  | 1.74 (1.21-2.61) | **4.21E-03** | **0.01** |  | 1.57 (1.08-2.39) | **0.02** | **0.05** |
| LAG-3 |  | 1.56 (1.10-2.29) | **0.02** | **0.02** |  | 1.52 (1.04-2.28) | **0.04** | **0.05** |

^a^ORs were estimated per 1 SD increase in log-transformed markers concentrations

^b^FDR-correction was applied

^c^Covariates included age, sex, smoking, BMI and diabetes

Significant *p* values in bold font

**Supplementary Table S3.** Characteristics of PDAC patients in the two soluble immune subtypes based on soluble immune checkpoint-related proteins.

| **Characteristics** | | **Cluster 1, n (%)** | **Cluster 2, n (%)** | ***p*** |
| --- | --- | --- | --- | --- |
| n |  | 33 | 37 |  |
| Subtype | | Soluble immune-high | Soluble immune-low |  |
| Age |  |  |  | 0.38 |
|  | <65 | 10 (30.30) | 16 (43.24) |  |
|  | >=65 | 23 (69.70) | 21 (56.76) |  |
| Sex |  |  |  | 0.67 |
|  | Female | 16 (48.48) | 15 (40.54) |  |
|  | Male | 17 (51.52) | 22 (59.46) |  |
| BMI |  |  |  | 0.31 |
|  | <25 | 26 (78.79) | 24 (64.86) |  |
|  | >=25 | 7 (21.21) | 13 (35.14) |  |
| Diabetes | |  |  | 0.86 |
|  | No | 23 (69.70) | 24 (64.86) |  |
|  | Yes | 10 (30.30) | 13 (35.14) |  |
| Smoke | |  |  | 0.31 |
|  | No | 21 (63.64) | 18 (48.65) |  |
|  | Yes | 12 (36.36) | 19 (51.35) |  |
| CA19-9 (U/ml) | |  |  | 0.79 |
|  | Normal (<37 U/ml) | 9 (27.27) | 8 (21.62) |  |
|  | Elevated (≥37 U/ml) | 24 (72.73) | 29 (78.38) |  |
| Tumor location | |  |  | 0.28 |
|  | Head | 20 (60.61) | 19 (51.35) |  |
|  | Neck | 3 (9.09) | 2 (5.41) |  |
|  | Body | 4 (12.12) | 2 (5.41) |  |
|  | Tail | 6 (18.18) | 14 (37.84) |  |
| T stage | |  |  | 0.81 |
|  | T1 | 1 (3.03) | 1 (2.70) |  |
|  | T2 | 11 (33.33) | 16 (43.24) |  |
|  | T3 | 11 (33.33) | 12 (32.43) |  |
|  | T4 | 10 (30.30) | 8 (21.62) |  |
| N stage | |  |  | 0.12 |
|  | N0 | 15 (45.45) | 24 (64.86) |  |
|  | N1 | 7 (21.21) | 8 (21.62) |  |
|  | N2 | 11 (33.33) | 5 (13.51) |  |
| M stage | |  |  | 0.10 |
|  | M0 | 25 (75.76) | 20 (54.05) |  |
|  | M1 | 8 (24.24) | 17 (45.95) |  |
| Stage | |  |  | 0.11 |
|  | Resectable | 14 (42.42) | 14 (37.84) |  |
|  | Locally advanced | 11 (33.33) | 6 (16.22) |  |
|  | Metastatic | 8 (24.24) | 17 (45.95) |  |
| Vital status | |  |  | **0.02** |
|  | Alive | 12 (36.36) | 25 (67.57) |  |
|  | Dead | 21 (63.64) | 12 (32.43) |  |

Significant *p* values in bold font

**Supplementary Table S4.** Characteristics of PDAC patients in TCGA-PAAD cohort.

|  |  | **PDAC, n (%)** |
| --- | --- | --- |
| n |  | 146 |
| Age, mean (SD) | | 64.77 (10.88) |
| Sex |  |  |
|  | Female | 68 (46.58) |
|  | Male | 78 (53.42) |
| Smoking | |  |
|  | No | 27 (18.49) |
|  | Yes | 119 (81.51) |
| Diabetes | |  |
|  | No | 88 (60.27) |
|  | Yes | 33 (22.60) |
|  | NA | 25 (17.12) |
| T stage | |  |
|  | T1 | 4 (2.74) |
|  | T2 | 16 (10.96) |
|  | T3 | 123 (84.25) |
|  | T4 | 3 (2.05) |
| N stage | |  |
|  | N0 | 36 (24.66) |
|  | N1 | 109 (74.66) |
|  | NX | 1 (0.68) |
| M stage | |  |
|  | M0 | 70 (47.95) |
|  | M1 | 3 (2.05) |
|  | MX | 73 (50.00) |
| Stage |  |  |
|  | I | 12 (8.22) |
|  | II | 128 (87.67) |
|  | III | 3 (2.05) |
|  | IV | 3 (2.05) |
| Grade |  |  |
|  | G1 | 20 (13.70) |
|  | G2 | 84 (57.53) |
|  | G3 | 41 (28.08) |
|  | G4 | 1 (0.68) |
| Vital status | |  |
|  | Alive | 90 (61.64) |
|  | Dead | 56 (38.36) |

**Supplementary Table S5.** Characteristics of PDAC patients in the two immune subtypes based on immune checkpoint genes.

| **Characteristics** | | **Cluster 1, n (%)** | **Cluster 2, n (%)** | ***p*** |
| --- | --- | --- | --- | --- |
| n |  | 95 | 51 |  |
| Subtype |  | Immune-low | Immune-high |  |
| Age |  |  |  | 0.33 |
|  | <65 | 39 (41.05) | 26 (50.98) |  |
|  | >=65 | 56 (58.95) | 25 (49.02) |  |
| Sex |  |  |  | 0.80 |
|  | Female | 43 (45.26) | 25 (49.02) |  |
|  | Male | 52 (54.74) | 26 (50.98) |  |
| Smoke |  |  |  | **0.02** |
|  | No | 12 (12.63) | 15 (29.41) |  |
|  | Yes | 83 (87.37) | 36 (70.59) |  |
| Diabetes |  |  |  | **0.02** |
|  | No | 66 (69.47) | 22 (43.14) |  |
|  | Yes | 17 (17.89) | 16 (31.37) |  |
|  | NA | 12 (12.64) | 13 (25.49) |  |
| T stage |  |  |  | 0.86 |
|  | T1 | 3 (3.16) | 1 (1.96) |  |
|  | T2 | 9 (9.47) | 7 (13.73) |  |
|  | T3 | 81 (85.26) | 42 (82.35) |  |
|  | T4 | 2 (2.11) | 1 (1.96) |  |
| N stage |  |  |  | 0.74 |
|  | N0 | 24 (25.26) | 12 (23.53) |  |
|  | N1 | 70 (73.68) | 39 (76.47) |  |
|  | NX | 1 (1.05) | 0 (0.00) |  |
| M stage |  |  |  | 0.67 |
|  | M0 | 43 (45.26) | 27 (52.94) |  |
|  | M1 | 2 (2.11) | 1 (1.96) |  |
|  | MX | 50 (52.63) | 23 (45.10) |  |
| Stage |  |  |  | 0.97 |
|  | I | 7 (7.37) | 5 (9.80) |  |
|  | II | 84 (88.42) | 44 (86.27) |  |
|  | III | 2 (2.11) | 1 (1.96) |  |
|  | IV | 2 (2.11) | 1 (1.96) |  |
| Grade |  |  |  | 0.83 |
|  | G1 | 13 (13.68) | 7 (13.73) |  |
|  | G2 | 53 (55.79) | 31 (60.78) |  |
|  | G3 | 28 (29.47) | 13 (25.49) |  |
|  | G4 | 1 (1.05) | 0 (0.00) |  |
| Vital status | |  |  | **0.01** |
|  | Alive | 51 (53.68) | 39 (76.47) |  |
|  | Dead | 44 (46.32) | 12 (23.53) |  |

Significant *p* values in bold font
